# Is Visual Selective Attention in Deaf Individuals Enhanced or Deficient? The Case of the Useful Field of View

**DOI:** 10.1371/journal.pone.0005640

**Published:** 2009-05-20

**Authors:** Matthew W. G. Dye, Peter C. Hauser, Daphne Bavelier

**Affiliations:** 1 Department of Brain and Cognitive Sciences, University of Rochester, Rochester, New York, United States of America; 2 Department of Research and Teacher Education, National Technical Institute for the Deaf, Rochester Institute of Technology, Rochester, New York, United States of America; National Institute of Mental Health, United States of America

## Abstract

**Background:**

Early deafness leads to enhanced attention in the visual periphery. Yet, whether this enhancement confers advantages in everyday life remains unknown, as deaf individuals have been shown to be more distracted by irrelevant information in the periphery than their hearing peers. Here, we show that, in a complex attentional task, a performance advantage results for deaf individuals.

**Methodology/Principal Findings:**

We employed the Useful Field of View (UFOV) which requires central target identification concurrent with peripheral target localization in the presence of distractors – a divided, selective attention task. First, the comparison of deaf and hearing adults with or without sign language skills establishes that deafness and not sign language use drives UFOV enhancement. Second, UFOV performance was enhanced in deaf children, but only after 11 years of age.

**Conclusions/Significance:**

This work demonstrates that, following early auditory deprivation, visual attention resources toward the periphery slowly get augmented to eventually result in a clear behavioral advantage by pre-adolescence on a selective visual attention task.

## Introduction

Several studies have demonstrated that early auditory deprivation (deafness) results in specific, compensatory changes in visual processing. In particular, deaf individuals exhibit enhanced performance for tasks performed in the visual periphery. Accordingly deaf individuals asked to make a key press in response to stimuli presented in the central or peripheral visual field, exhibit faster RTs than hearing individuals for peripheral targets but not for central ones [1]–[3]. Similarly, when required to indicate the presence of a point of light moving from the periphery towards fixation, they respond more accurately than hearing individuals when the target is further away from fixation [4]. Brain imaging studies using ERP or fMRI suggest a greater recruitment of attention-related brain networks under peripheral tasks in deaf as compared to hearing individuals [5]–[9].

Whether this enhancement confers advantages when it comes to more complex visual tasks is, as yet, unknown. We consider here the possibility that enhanced peripheral attention may result in better or worse performance depending upon the task demands. Deaf individuals are more distracted than their hearing peers by irrelevant information occurring in the visual periphery [10]–[12]. This effect is not the mark of a deficient focus of attention in deaf individuals – indeed, hearing individuals are more distracted than their deaf peers by irrelevant information occurring in central vision. Yet, greater distractibility from peripheral events can be disruptive when focusing centrally is required [13]. Accordingly, deaf children perform worse on the Gordon Diagnostic System®, a continuous performance task which measures the ability to select a sequence of targets from a stream of items presented in central vision [14]–[15]. In accordance with a deficiency hypothesis, deaf children are rated more distractible than their hearing peers by parents and educators, although the correlation between these ratings is often low [16]. Based on these findings, it has been argued that auditory deprivation results in deficient visual selective attention, with deaf individuals being unable to differentiate task-relevant from task-irrelevant information [16]. In contrast, as we explore here, deaf individuals may excel on tasks that require differentiating task-relevant from task-irrelevant information when this selection relies on peripheral visual attention.

The performance of deaf and hearing individuals on a computerized adaptation of the Useful Field of View task (UFOV; [17]–[18]) was evaluated. In this task, subjects are required to identify a central target and localize a concurrent peripheral target in the presence of distractors. Performance on the UFOV – which is predictive of complex, real-world performance [19] – provides a measure of how visual selective attention is distributed across a scene when attention has to be allocated across central and peripheral locations and targets selected from within a field of distractors. If early auditory deprivation enhances visual selective attention resources in the periphery, rather than simply increasing peripheral distractibility, then deaf individuals should be better able to localize a peripheral target embedded in a field of distractors while simultaneously discriminating the identity of a target presented centrally at fixation. Alternatively, if auditory deprivation results in deficient visual selective attention, deaf individuals' performance on the UFOV task should be impaired relative to that of hearing individuals.

The majority of studies reporting enhancement of visual attention to the periphery have recruited deaf individuals born deaf to deaf parents who learned American Sign Language (ASL) as a first language. This leaves open the possibility that enhancements in attention are restricted to this sub-population and do not generalize to the deaf population at large. This is of concern as most studies reporting deficient visual attention have focused on deaf non-signers. Therefore, in addition to recruiting deaf native signers, we included deaf individuals who experienced early auditory deprivation but did not learn a sign language. In addition, the impact of sign language was further evaluated using hearing subjects, born to deaf parents, who acquired ASL as a first language. Some of the studies referenced above have included hearing native signers, and have suggested that sign language use is not sufficient to induce enhanced peripheral attention [20]–[22]. The possibility remains, however, that a combination of early auditory deprivation and visual-manual language acquisition are required to bring about the observed changes in peripheral attention in deaf native signers. The inclusion of both hearing signers and deaf non-signers allows, for the first time, an assessment of the effects of auditory deprivation and sign language use independently, as well as their potential interaction.

## Materials and Methods

### Ethics Statement

This research was approved by the Research Subjects Review Board at the University of Rochester, NY.

### Design

**Figure 1:**
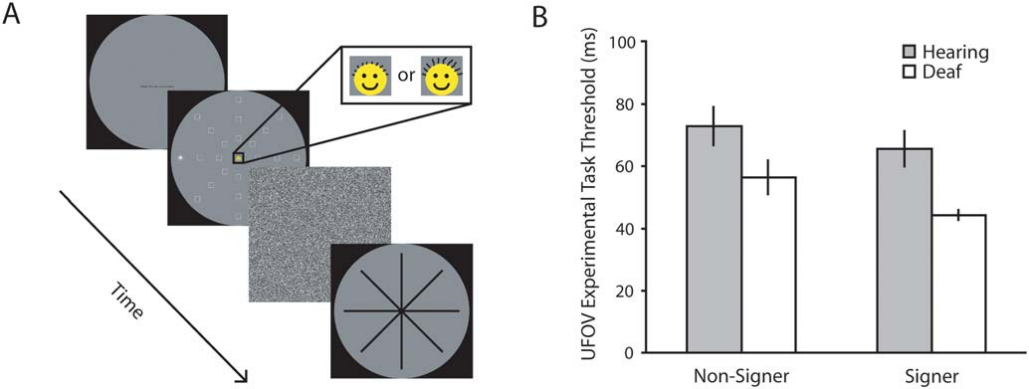
A Schematic of Useful Field of View Task. In the experimental UFOV task, participants were asked to discriminate a briefly presented face in the center of the display – the cutaways show detail of the ‘short hair’ and ‘long hair’ faces – and to indicate the location of a peripheral target (a five-pointed star in a circle) via a touch screen. B Useful Field of View Thresholds, Experiment 1. Performance (mean threshold is ms) of each subject group on the experimental UFOV task; error bars indicate ±1 SEM.

Each subject was assessed using a modification of the UFOV paradigm [17]–[18], which incorporated two training tasks and the main experimental task (see Figure 1A) administered in the following order: (i) central stimulus identification training: a two-alternative forced choice (2-AFC) identification task at the center of the visual field – a face icon subtending 2.0 degrees of visual angle was presented in the center of the screen and participants had to decide whether it had long (0.27 degrees of visual angle) or short (0.16 degrees of visual angle) hair (Movie S1); (ii) divided attention training task: the same 2-AFC central identification task combined with the localization of a peripheral target (again subtending 2.0 degrees of visual angle) presented in isolation at 20° of visual angle at one of eight possible cardinal/intercardinal locations (Movie S2); and (iii) the UFOV experimental task, also termed selective attention task by Ball and collaborators [17]–[18]: the 2-AFC central identification task with localization of a peripheral target always presented at 20° of visual angle at one of eight possible cardinal/intercardinal locations and embedded in a field of 27 distractors each subtending 2.0 degrees of visual angle (Movie S3). The distractors appeared along each of the eight possible cardinal/intercardinal axes at 6.67, 13.33 and 20 degrees of visual angle (see Figure 1A). The peripheral target was a five-pointed star enclosed within a circle, and the distractors were white line-drawings of squares isoluminant with the peripheral target.

All three tasks were presented within a circular gray field subtending 21° of visual angle. Each stimulus display was followed by a visual noise mask presented on the whole screen and then a prompt appearing at fixation. Participants indicated their response (in speech or sign) for the central task, and the experimenter manually entered that response. For the peripheral localization response, participants touched the screen at the location where they believed the peripheral target to have appeared. Trials were classified as correct if the subject accurately identified both the central icon's identity and the location of the peripheral target (in the first task, only central task performance applied). An adaptive staircase procedure was employed for all three tasks – after three consecutive correct responses, the stimulus duration was reduced by 1 frame (1/60 second); one incorrect response resulted in the stimulus duration being increased by 1 frame. Each task finished after twelve reversals, ten consecutive correct trials at ceiling (1 frame), or 72 trials, whichever occurred sooner. A threshold measure was calculated by averaging the stimulus duration of the last 10 correct trials. In the divided attention training and the UFOV tasks – which required both central and peripheral responses – trials where the central target was incorrectly identified were ignored (i.e. those trials were not used for computing step changes in the adaptive staircase procedure).

### Apparatus

Stimuli were presented using Matlab software and the Psychophysics Toolbox installed on a Apple G4 Titanium laptop computer running OS 9.2.2. The laptop was connected to a 23″ Apple Cinema Display via an Apple ADC-DVI adaptor, with a 60 Hz refresh rate. The display was adapted to function as a touch screen using pressure-sensitive resistive (PSR-1®) technology, supplied and fitted by Troll Touch Touchscreens (Valencia, CA).

### Procedure

Subjects were tested in a single experimental session lasting approximately 25–30 minutes. Subjects were in a chin rest, positioned 40 centimeters from the center of the touch screen. Instructions were given in sign or speech and clarified if necessary. Subjects were given the correct answer on the first 2–3 trials if they still appeared to be confused.

## Results and Discussion

All statistical tests were conducted with an α = .05. Confidence intervals for differences between group means (CI95_diff_) are reported alongside statistical test results and estimates of effect size (partial η^2^).

### Experiment 1: Effects of Deafness and Sign Language Experience on the Useful Field of View in Adults

Potential adult subjects were asked about their videogame playing. Those who reported playing action-based videogames were classified as ‘game players’. This classification did not influence enrollment into the study, although data from ‘game players’ are not reported here as it is known that action video gaming changes performance on the UFOV [23]–[24]. Subjects were paid $8 for their participation.

Deaf adult signers (N = 10, M_AGE_ = 26.1, 2 males) were recruited at a school in Austin (TX) and at a camp in Madison (SD), as well as from participant pools at RIT/NTID (NY) and Gallaudet University (DC). All were deaf native signers who reported being born with severe-profound auditory deprivation (hearing loss >75 dB in the better ear; for 5 deaf signers who knew their exact level of hearing loss, mean loss in the better ear was 107 dB with a range of 75–120 dB) to deaf parents from whom they learned ASL as a first language. In the absence of a reliable and easily administered ASL proficiency test, subjects were asked to rate their ASL comprehension and production proficiency on a scale from 1 = perfect to 4 = hardly. All deaf signers gave themselves a rating of 1.0 in ASL comprehension and 1.0 in ASL production.

Deaf adult non-signers (N = 10, M_AGE_ = 21.6, 3 males) were students recruited at the National Technical Institute for the Deaf (NTID) in Rochester, NY. All reported being born with severe-profound auditory deprivation (>75 DB in the better ear; for 6 deaf non-signers who knew their exact level of hearing loss, mean loss in the better ear was 90 dB with a range of 75–110 dB). Although most reported knowing some ASL, their first regular exposure to ASL had been at NTID where they were recruited for this study during their first quarter in order to limit that exposure. Accordingly, they reported an inability to communicate clearly in ASL (on average rating themselves 3.3 in ASL comprehension and 3.2 in ASL production). All deaf non-signing subjects preferred testing to be conducted using spoken English.

Hearing adult signers born to deaf parents (N = 10, M_AGE_ = 22.9, 4 males) were recruited from a summer camp for KODAs (‘kids of deaf adults’) in Eagle Bay, NY. All reported learning ASL from their parents as infants, and expressed competence in ASL (on average rating themselves 1.8 in ASL comprehension and 1.8 in ASL production). None reported any hearing loss, and all testing was conducted in ASL.

Hearing adult non-signers (N = 10, M_AGE_ = 20.4, 2 males) were recruited from a participant pool at the University of Rochester, NY. All reported normal hearing and no knowledge of any sign language.

Prior to analyzing the UFOV thresholds for the selective attention task (i.e. the task with distractors) it was important to establish that the central task was attentionally demanding in this context, and thus in competition with the peripheral target for attentional resources. While this task provided no independent, concurrent measure of central task performance, identification accuracy was calculated for the last 1/3 of trials for all subjects (see Table S1). Due to differences in level of performance – these trials for deaf subjects were performed at briefer presentation durations than for hearing subjects –identification accuracies were normalized as a function of the presentation duration for those trials to yield a measure of central task accuracy per millisecond of presentation duration. Deaf subjects (M = 1.46% per millisecond) and hearing subjects (M = 1.25% per millisecond) did not significantly differ using this measure. The data therefore suggest that the central task was attentionally demanding for both deaf and hearing subjects, and that it was equally demanding for both groups.

UFOV thresholds (i.e. with distractors present) were entered into a two-way ANOVA with auditory deprivation (deaf, hearing) and signing (signer, non-signer) as between subjects factors (see Figure 1B). This revealed a main effect of auditory deprivation (F(1,36) = 11.46, p = .002, partial η^2^ = .24, CI95_diff_ = 8–30 ms). Deaf subjects demonstrated a clear advantage over hearing subjects, requiring less time to concurrently discriminate a central target and localize a peripheral target embedded within a field of distractors. An effect of sign language use was not predicted, and although a trend can be seen for sign language users to have lower thresholds than non-signers, the effect was much smaller and not statistically significant (F(1,36) = 3.02, p = .091, partial η^2^ = .08, CI95_diff_ = 2–21 ms). There was no significant interaction between auditory deprivation and signing (F(1,36) = 0.18, p = .677, partial η^2^ = .01) confirming the primary role of auditory deprivation in the advantage noted in the deaf population.

**Figure 2:**
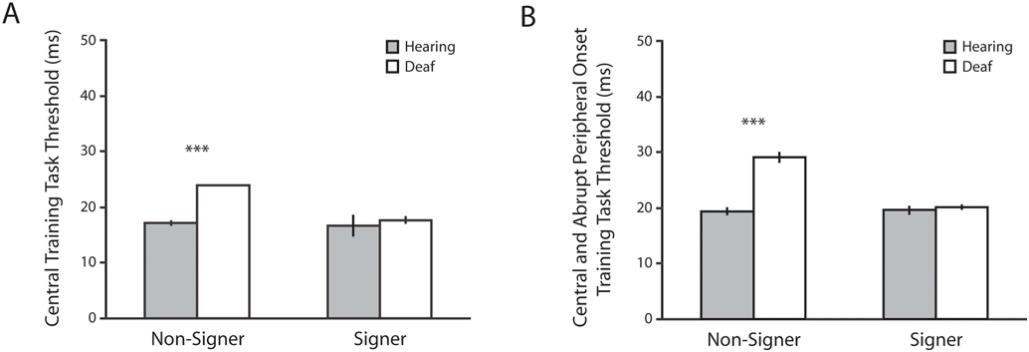
Useful Field of View Training Thresholds, Experiment 1. Performance on the central training task (A) and central and abrupt peripheral onset training task (B) was generally asymptotic, except for deaf adults who did not use a signed language. For this group, the thresholds on these two tasks were significantly elevated. Error bars indicate ±1 SEM.

Although the two other tasks (central stimulus identification and divided attention) were included for training purposes, deaf non-signer participants differed from the other groups in a manner worthy of note (Figure 2A and 2B). On both tasks, all participants performed near ceiling except for deaf non-signers (central identification task: effect of auditory deprivation: F(1,36) = 12.51, p = .001, partial η^2^ = .26, CI95_diff_ = 2–6 ms; effect of signing: F(1,36) = 9.52, p = .004, partial η^2^ = .21, CI95_diff_ = 1–6 ms; interaction between auditory deprivation and signing: F(1,36) = 6.94, p = .012, partial η^2^ = .16; divided attention task: effect of auditory deprivation: F(1,36) = 41.40, p<.001, partial η^2^ = .54, CI95_diff_ = 3–7 ms; effect of signing: (F(1,36) = 31.25, p<.001, partial η^2^ = .47, CI95_diff_ = 3–6 ms; interaction between auditory deprivation and signing: F(1,36) = 33.65, p<.001, partial η^2^ = .48). Deaf non-signers performed significantly worse on both of these tasks, albeit still requiring less than 33 milliseconds of presentation. This is in accord with reports from Quittner and colleagues [14]–[15] that deaf individuals, or at least children, who do not receive full access to language at an early age are at risk on tasks that require attention to the location of fixation.

UFOV thresholds were reanalyzed with each subject's performance on these training tasks as covariates. The pattern of findings did not change, with the main effect of auditory deprivation remaining the sole significant effect (F(1,34) = 6.21, p = .018, partial η^2^ = .15, CI95_diff_ = 4–39 ms).

This first experiment establishes the role of auditory deprivation in the enhancement of peripheral visual attention noted in the deaf population. Both deaf signing and deaf non-signing adults excelled at the UFOV task. This shows that the enhancement is not limited to the use of isolated targets but generalizes to complex tasks such as the UFOV, which combines selective visual attention with the requirements of performing two tasks (one centrally and the other peripherally). Although deaf non-signers displayed better performance on the UFOV task than their hearing peers, they showed worse performance on the central stimulus identification and divided attention tasks. This result is surprising in the face of their enhanced performance on the UFOV task. The two training tasks differ from the main UFOV task along several dimensions preventing us from drawing firm conclusions. The central identification task focuses entirely on central processing, rather than peripheral processing in the context of an additional central task like in the UFOV task. The divided attention task requires both peripheral and central processing in the same manner as for the UFOV task, but it differs from the UFOV task in terms of its very low attentional load [25]; the divided attention task allows both the central and peripheral target to automatically capture attention. In addition both these training tasks differ from the UFOV task in the brevity of the display duration (stimulus display durations for the two training tasks were in the range of 17–33 ms, as compared to 40–80 ms for UFOV task). The reported results indicate the need for future studies to characterize the relative role of central processing, attentional load and display duration when considering the attentional system of deaf non-signers.

There are two alternative mechanisms that can be ruled out by the overall pattern of data reported. The first is that any deficits observed for deaf individuals stem from the need to make sequential manual responses (sign SHORT or LONG and then touch the screen) whereas hearing individuals can make a simultaneous oral-manual response (say “short” or “long” while touching the screen at the same time). If this were the case, then there should be a deficit for deaf signers across all tasks requiring two responses, which is clearly not the case. Despite the need to execute sequential responses for the two tasks, deaf signers outperform hearing subjects on the UFOV task, and show comparable performance on the divided attention task. Indeed, the deaf non-signers who performed poorly on the divided attention task made simultaneous oral and manual responses to the targets. The second alternative is a perceptual enhancement in the peripheral visual field of deaf individuals. Such an enhancement would predict enhanced performance on the divided attention task for all deaf individuals. To the contrary, deaf non-signers showed impaired performance on the divided attention task and deaf signers showed similar performance as their hearing peers. This pattern of finding reinforces the view that peripheral processing enhancements in deaf individuals result from changes in selective attention, and not perceptual modifications [26].

In Experiment 2 we ask at what age such a redistribution of attention becomes apparent in a sample of deaf children compared to a group of hearing peers 7 to 17 years of age. Deaf children were recruited from a camp and deaf school where ASL was the primary means of communication. The experimental design, apparatus and procedure were the same as those employed in Experiment 1. Previous studies suggest that visual selective attention skills are relatively stable in hearing subjects by 7–10 years of age [27], so no change in the UFOV thresholds was expected in the hearing children as a function of age. By assessing the effect of age on UFOV thresholds in deaf children, we aimed to determine whether the effects of auditory deprivation on visual selective attention were already in place by the age of 7 years, or whether the period of development is protracted.

### Experiment 2: Effects of Deafness on the Useful Field of View in Deaf and Hearing Schoolchildren

Written informed consent was obtained from all children and a parent or legal guardian. All children were rewarded with a $15 gift card. As in Experiment 1, action video game players were tested but their data are not reported here.

Hearing children were recruited from a school district in the Rochester NY area. Mailings were sent from the school district to parents of all children aged 7 to 17 years. The response rate was approximately 15%. All children had normal or corrected-to-normal vision and no known history of neurological or cognitive impairment. They were screened to ensure none required an Individualized Educational Program (IEP) indicating the need for accommodations due to learning or language impairment. Children were divided into three age categories: 7–10 year old elementary/primary students (N = 38, M_AGE_ = 9;1, 16 males), 11–13 year old middle school students (N = 16, M_AGE_ = 12;2, 5 males), and 14–17 year old high school students (N = 14, M_AGE_ = 15;7, 1 male).

Deaf children were recruited from deaf schools in Rochester NY and Austin TX, and a camp for deaf children in Madison, SD. School or camp administrators mailed letters to the parents of all children aged between 7 and 17 years, resulting in a 10% response rate. All children had normal or corrected-to-normal vision and no known history of neurological or cognitive impairment. Although most of the deaf children had IEPs as a result of their deafness, none had reported attentional problems or learning disabilities. The deaf children divided into the same age categories as hearing children: 7–10 year olds (N = 15, M_AGE_ = 9;3, 10 males), 11–13 year olds (N = 20, M_AGE_ = 12;4, 1 male), and 14–17 year olds (N = 14, M_AGE_ = 15;6, 7 males). All had an unaided hearing loss >70 dB in their better ear and used ASL on a daily basis as their primary means of communication. None had undergone cochlear implant surgery. Sixteen (33%) had hearing parents, although all of these children had started to learn ASL in pre-K classes. Parental hearing status had no effect on the measures used, and is not considered further. Given their background, this group is more similar to the native signers adults described above, and differs in aetiology from the children typically considered in the literature on deafness, visual attention and cochlear implants [14]–[16].

**Figure 3:**
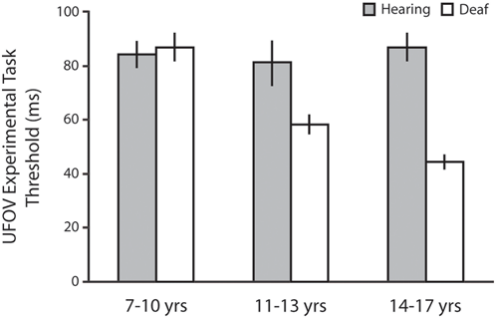
Useful Field of View Thresholds, Experiment 2. In the main UFOV task the performance of 7–10 year old deaf children was comparable with that of their hearing peers, whereas older deaf children were significantly better than their hearing peers. Error bars indicate ±1 SEM.

A two-way ANOVA on experimental UFOV thresholds with auditory deprivation (deaf, hearing) and age group (7–10, 11–13, 14–17 years) as between subjects factors revealed significant main effects of auditory deprivation (F(1,117) = 17.85, p<.001, partial η^2^ = .14, CI95_diff_ = 11–31 ms) and age group (F(2,117) = 6.49, p = .002, partial η^2^ = .11), and a significant two-way interaction between auditory deprivation and age group (F(2,117) = 7.10, p = .001, partial η^2^ = .11). This interaction led us to assess the effects of age group separately for deaf and hearing children. As predicted, for hearing children the UFOV thresholds did not vary as a function of age group (F(2,68) = 0.12, p = .884, partial η^2^<.01), whereas they did for deaf children (F(2,49) = 25.43, p<.001, partial η^2^ = .53). While deaf 7–10 year olds performed equivalently to hearing 7–10 year olds, older deaf children demonstrated better thresholds, outperforming their hearing peers and the youngest deaf children (see Figure 3).

**Figure 4:**
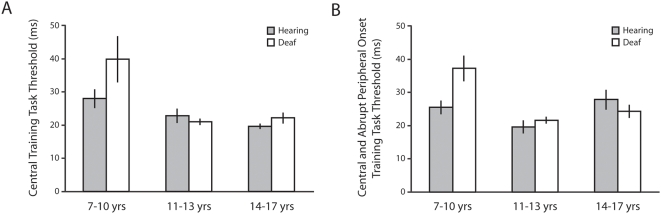
Useful Field of View Training Thresholds, Experiment 2. On the two training tasks, the youngest deaf children (7–10 year olds) performing significantly worse than their hearing peers. Across all age ranges tested, for both deaf and hearing samples, these children were the only ones who did not perform near ceiling on these tasks.

Interestingly, the training tasks indicated worse performance in the youngest deaf group compared to the other groups (see Figure 4). Post-hoc analyses for the central stimulus identification task showed no main effect of age group for hearing children (F(2,68) = 2.26, p = .113, partial η^2^ = .07), but a significant effect for deaf children (F(2,49) = 11.87, p<.001, partial η^2^ = .34). Deaf 7–10 year olds had significantly worse thresholds than both 11–13 year olds (p<.001, CI95_diff_ = 9–22 ms) and 14–17 year olds (p = .001, CI95_diff_ = 6–20 ms). Similarly, post-hoc analyses for the divided attention task showed no significant main effect of age group for hearing children (F(2,68) = 2.09, p = .132, partial η^2^ = .06), whereas it did significantly affect the performance of deaf children (F(2,49) = 7.17, p = .002, partial η^2^ = .24). As observed for the central identification task, deaf 7–10 year olds had significantly worse thresholds than 11–13 (p = .001, CI95_diff_ = 8–30 ms) and 14–17 year olds (p = .004, CI95_diff_ = 6–29 ms). Using performance on the two training tasks as covariates did not change the qualitative pattern of findings for the experimental UFOV task.

### General Discussion

The UFOV task requires subjects to divide attention between central and peripheral locations, while also selecting a target from amongst distractors. It is an attention-demanding task, requiring not only central attention but also attention to the periphery and visual selection. Deaf adults required 43–58 ms (CI95%) of display presentation to perform at 79% correct, whereas hearing adults required significantly more time (CI95% = 60–79 ms). This enhancement reflects early, severe-profound loss of audition rather than use of a visual-spatial language – the effect was seen in both deaf signers and non-signers, with little-or-no contribution from signing.

Although a tendency for more effective visual search in deaf than in hearing individuals has been reported [28], other studies have failed to replicate the effect [29]–[30]. The present adaptation of the UFOV task departs from these more standard visual search tasks in several ways. First, while it maintains a requirement for visual selection, it also has a divided attention component where attention needs to be maintained centrally while also efficiently allocated to the periphery. Auditory deprivation may thus enhance the ability to deploy visual selective attention over a large field. Second, the target to be selected needs to be localized rather than identified. The use of a touch screen ensures that localization information maps naturally onto a motor response, limiting the need to repackage the information as with a standard response box. This makes for a very natural “where” task, in line with the proposal that dorsal visual functions are most likely to be enhanced following auditory deprivation [31]. These factors may account for the sizeable advantage noted in the deaf population, revealed by both lower thresholds and smaller within-group variance. We propose that the UFOV task data unambiguously make the case that auditory deprivation does not necessarily compromise visual selective attentional functions and can in fact result in enhanced selection for stimuli presented peripherally.

Data from children revealed that this attentional enhancement is not observed until after 7–10 years of age, although the precise point within this age group could not be determined due to sample size limitations. Nevertheless, there is the suggestion that a robust cross-modal enhancement in visual selective attention is not observed until after several years of auditory loss. Further study is required to identify exactly when and how this delayed enhancement is brought about. The lack of improvement observed in hearing children suggests that maturational factors are unlikely to contribute. Rather, it may be the duration of auditory deprivation that plays the key role, with over 7–10 years of auditory deprivation required for effects to be manifested behaviorally. Alternatively, it may reflect a ‘sleeper effect’ [32], with significant neural changes occurring earlier in development, but only manifesting themselves behaviorally at a later age. Another possibility is that the reorganization of visual attention is trigged by environmental stimuli. For example, the transition to more formal and structured schooling environments around the age of 8 years may place additional demands upon the visual systems of deaf children. Assessment of this possibility will require disentangling duration of deafness from educational experience; all of the children included in this study were born deaf and thus duration of deafness and level of schooling are confounded. For now, the data provide evidence for a profound change in visual selective attention in deaf children and adults, with robust effects suggesting that the UFOV is a sensitive behavioral assay for further analysis of the causes and mechanisms of compensatory, cross-modal plasticity following early auditory deprivation.

There is some evidence that the youngest deaf children found the central stimulus identification task to be more difficult than did their hearing peers, with this difficulty extending to the divided attention task. Data from deaf adults suggest that any such deficit is no longer apparent by adulthood, at least for those who have early and full access to a first language (deaf native signers). All of the young deaf children in this study had early language access through ASL, although the extent of their social and linguistic interactions with caregivers during early infancy cannot be assessed post hoc. It is important to note that studies reporting deficiencies in visual attention skills have typically used central visual field tasks employing rapid stimulus presentations with young deaf children [14]–[15], and those reporting compensatory enhancements have used peripheral visual field tasks with deaf adults [2], [4], [10]–[12]. Thus the apparent discrepancy in the literature may be due to cross-study differences in the age of subjects tested, language history, and where in the visual field stimuli have been presented. For both deaf children under the age of 10 years and deaf adults who have had delayed and impaired access to a first language, the present work highlights poorer performance on the two training tasks alongside enhanced UFOV performance. The present design cannot distinguish between a possible central processing disadvantage when attention is not heavily taxed or a difficulty processing displays with very brief durations. Future research is needed to tease apart the relative role of central versus peripheral attentional demands and to evaluate processing of very brief displays in deaf individuals. This work, however, already highlights the importance of providing a strong language environment early in development. By 11 years of age, the performance of deaf native signers was equal to or better than their hearing peers on all tasks, whereas deaf non-signers still exhibited a complex pattern of deficits and enhancements in adulthood. Finally and most importantly, a robust advantage for all deaf populations was observed when the peripheral target had to be selected from amongst distractors, paralleling findings reported by others [33]. The addition of distractors changes the task by requiring coupling of divided attention with efficient visual selective attention at the target location. It is under these conditions – visual selective attention in the visual periphery – that deaf participants are seen to excel.

This work establishes that auditory deprivation is not a causal factor for attentional difficulties. All deaf individuals tested performed at least as well and often significantly better than their hearing peers on the UFOV measure, an attentionally-demanding task Worse performance in the youngest deaf children and those deaf adults with limited access to a natural language early in development was noted under some conditions. While these results are in line with previous work documenting attentional deficits in deaf children, the present study makes it clear that such challenges early in childhood are not predictive of deficient functioning as development proceeds.

## Supporting Information

Table S1 Central task performance in selective UFOV task. For the UFOV selective attention task, mean central identification accuracies and mean stimulus presentation durations were calculated based upon the last 1/3 of trials for each subject. Accuracy levels indicate that the central task is attentionally demanding for all subject groups. However, accuracy cannot be compared directly across groups, as presentation durations differed. After normalizing accuracies as a function of presentation duration, performance did not significantly differ as a result of deafness or sign language use.
(0.04 MB DOC)

Movie S1 Two-alternative forced choice (2-AFC) discrimination task at the center of the visual field - a face icon was presented in the center of the screen and participants had to decide whether it had long or short hair.
(0.42 MB MOV)

Movie S2 The same 2-AFC central discrimination task as in Movie S1, combined with the localization of a peripheral target presented in isolation at 20° of visual angle at one of eight possible cardinal/intercardinal locations
(0.62 MB MOV)

Movie S3 The 2-AFC central discrimination task with localization of a peripheral target presented at 20° of visual angle at one of eight possible cardinal/intercardinal locations and embedded in a field of distractors. The distractors appeared along the eight possible cardinal/intercardinal axes at 6.67, 13.33 and 20 degrees of visual angle.
(0.52 MB MOV)
