# Supplementary material for: Is Visual Selective Attention in Deaf Individuals Enhanced or Deficient? The Case of the Useful Field of View
Source: PLoS One. 2009 May 20;4(5):e5640. doi: 10.1371/journal.pone.0005640 (PMC2680667; doi:10.1371/journal.pone.0005640)
Supplement: Table S1 — Central task performance in selective UFOV task. For the UFOV selective attention task, mean central identification accuracies and mean stimulus presentation durations were calculated based upon the last 1/3 of trials for each subject. Accuracy levels indicate that the central task is attentionally demanding for all subject groups. However, accuracy cannot be compared directly across groups, as presentation durations differed. After normalizing accuracies as a function of presentation duration, performance did not significantly differ as a result of deafness or sign language use. (0.04 MB DOC) [file pone.0005640.s001.doc]

|  |  | *Central Identification Accuracy (%)* | *Stimulus Presentation Duration (ms)* | *Accuracy per Unit of Presentation Duration (%/ms)* |
| --- | --- | --- | --- | --- |
| Deaf | Signer | 85.9 | 58.3 | 1.47 |
|  | Non-Signer | 89.4 | 61.6 | 1.45 |
| Hearing | Signer | 96.7 | 75.0 | 1.29 |
|  | Non-Signer | 96.5 | 80.0 | 1.21 |

**Table S1. Central task performance in selective UFOV task.** For the UFOV selective attention task, mean central identification accuracies and mean stimulus presentation durations were calculated based upon the last 1/3 of trials for each subject. Accuracy levels indicate that the central task is attentionally demanding for all subject groups. However, accuracy cannot be compared directly across groups, as presentation durations differed. After normalizing accuracies as a function of presentation duration, performance did not significantly differ as a result of deafness or sign language use.
